# Radiation for Awakening the Dormant Immune System, a Promising Challenge to be Explored

**DOI:** 10.3389/fimmu.2014.00102

**Published:** 2014-03-14

**Authors:** Luis de la Cruz-Merino, Ana Illescas-Vacas, Ana Grueso-López, Antonio Barco-Sánchez, Carlos Míguez-Sánchez, Cancer Immunotherapies Spanish Group (GETICA)

**Affiliations:** ^1^Clinical Oncology Department, Virgen Macarena University Hospital, Seville, Spain; ^2^Radiotherapy Department, Virgen Macarena University Hospital, Seville, Spain; ^3^Biochemistry Department, Virgen Macarena University Hospital, Seville, Spain

**Keywords:** immunotherapy, radiotherapy effects, tumor microenvironment, abscopal effect, CTLA-4

## Abstract

Recent advances that have been made in our understanding of cancer biology and immunology show that infiltrated immune cells and cytokines in the tumor microenvironment may play different functions that appear tightly related to clinical outcomes. Strategies aimed at interfering with the cross-talk between microenvironment tumor cells and their cellular partners have been considered for the development of new immunotherapies. These novel therapies target different cell components of the tumor microenvironment and importantly, they may be coupled and boosted with classical treatments, such as radiotherapy. In this work, we try to summarize recent data on the microenvironment impact of radiation therapy, from pre-clinical research to the clinic, while taking into account that this new knowledge will probably translate into indication and objective of radiation therapy changes in the next future.

## Introduction

In the last few years, the impact of the specific immune microenvironment in cancer has gained renewed interest, and is actually recognized as one of the major determinants of clinical evolution in a wide range of tumors. In this sense, cells like tumor-associated macrophages (TAM), regulatory T cells (Treg), and myeloid-derived-suppressor cells (MDSC), among others, are being proposed as new prognostic biomarkers that might be taken into account for diagnostic purposes, with the aim to complete classical information that is generally focused mainly on intrinsic characteristics of the tumor cell itself (histopathologic grade, mitosis, etc). Importantly, the effect of antineoplastic treatments on tumor cells may change the composition of microenvironment cells and their functional status, which introduces even more complexity (and importance) to this topic.

Radiotherapy (RT) remains a cornerstone of oncological treatment for many types of tumors. Recently, it has been demonstrated that ionizing radiation may exert interesting effects over the tumor microenvironment, increasing the effectiveness of patients’ anti-tumor immune responses in the clinical setting even at distant sites (1). This fact has given rise to the concept of immunogenic death mediated by radiation, which seems largely associated with the immunocompetence status of the host (2).

In this review, we pursue to update and summarize the local and systemic immune effects of RT from the molecular level to the clinical scenario. Finally, some choices for immunotherapy combinatorial approaches based on RT and strategies for monitoring these sorts of response are suggested.

## Local Immune Effects of RT

The radiation-induced biological response exerts pro-inflammatory and immunomodulatory effects against tumor cells, and nowadays the modulation of the acquired immune response after irradiation is gaining increased interest (1).

The radiobiological model considers that DNA damage after radiation induces different types of biological response, this has been classically described as the 5 Rs of radiobiology (intrinsic radiosensitivity, reoxygenation, redistribution in the cell cycle, repair of sublethal damage, and accelerated repopulation) (3). In this model, the radiobiological effects are caused by direct damage on DNA by tumor cells or indirectly after the induction of free radicals. It is worthy of consideration that this fact may determine a variable type of cell death through the phenomena of apoptosis, autophagy, necrosis, or mitotic catastrophe (4). Most cells survive a limited period of time after irradiation and, during this time, they generate molecular signals that induce the overexpression of specific genes that control the expression of growth factors, cytokines, chemokines, and cell surface receptors. Cell survival depends on this response and its ability to repair damaged DNA, being these phenomena of primary importance in radiation treatments, as they may determine the final effects over the surrounding microenvironment (5).

Increasing evidence has revealed that RT can change its recognition level, making the tumor vulnerable to the immune system. Furthermore, it has been described that the radiobiological response causes the activation of different T-cell lines, generating the “switch-on” of the adaptive immune response (6). These findings have led the scientific community to explore the immunotherapy and the RT effects together, as synergic tools in cancer treatment strategies (7).

**Figure 1:**
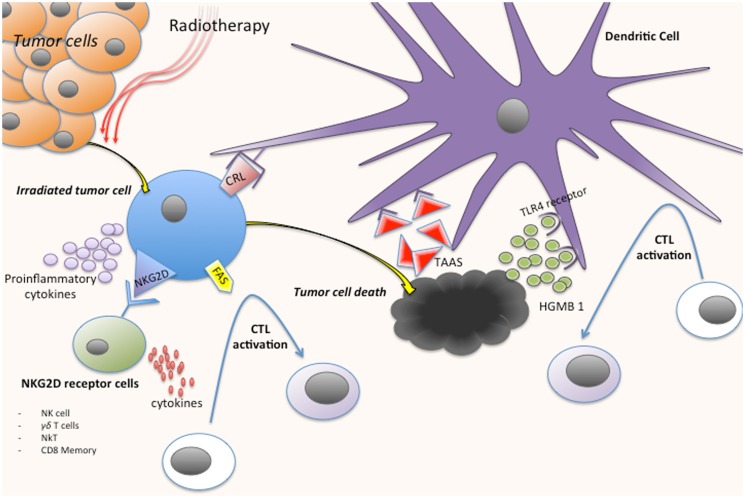
**Immune response activation process after tumor cells irradiation**. CRL, calreticulin; CTL, cytotoxic cell; TAAS, tumor-associated antigens; HMGB1, high-mobility group box 1.

It seems that one of the main effects of RT to unleash an effective immune response is the induction of a strong “danger signal,” which is a concept postulated by Polly Matzinger in 1994, related to the stress signals generated by the damaged tissue (8). Dying tumor cells after irradiation induce danger signals like endogenous ligands called “alarmins” with immunogenic properties. Various mechanisms with different peptides, cytokines, and cells are involved in this process (Figure 1):

(1) Calreticulin: Radiation causes the translocation of calreticulin (CRL) from the endoplastic reticulum to the cell surface, inducing the apoptotic cell antigen presentation to antigen presenting cells (APCs), in particular dendritic cells (DC), and stimulating specific anti-tumor T-cell responses (9, 10).
(2) High-mobility group box 1 (HMGB1): Another immunogenic determinant of cell death is the pro-inflammatory factor HMGB1. HMGB1 is a nuclear protein that is released after necrotic cell death and from dying cells during late stage apoptosis. After cell death induced by RT, HMGB1 may be released to the stroma and act as a neo-antigen, which in turn acts as an immunogenic endogenous “danger signal,” initiating an inflammatory response through binding Toll-Like Receptor 4 (TLR4) on DC (9, 11).
(3) NKG2D receptor: NKG2D acts as an activating receptor on NK cells, γδ T cells, NKT cells, and memory and activated CD8^+^ T cells (12). RT induces the expression of NKG2D ligands that, after engagement with its receptor, seems to increase cytokine production in order to stimulate CTL (12, 13).
(4) Upregulation of death receptors: RT may activate the extrinsic pathway of apoptosis, upregulating the expression of the FAS death receptor on tumor cells, which induces the activation of CTL via FAS ligand expressed on their surface (14).
(5) Release of tumor antigens: Apoptotic and necrotic tumor cells after RT are a big source of tumor antigens, commonly tumor-associated antigens (TAAs), which can be efficiently taken up by DC that subsequently present them to CTL (15) (this topic is amplified on section “Conclusion”).
(6) Release of pro-inflammatory cytokines: Pro-inflammatory cytokines like IL-1β, TNF-α, or prostaglandin E2 are up-regulated in tumor cells after the cellular lesion post-irradiation, and they represent ultimately danger signals due to the tissue stress induced by RT (16).

Immune-modulating effects of radiation are influenced by several factors. In this sense, the dose of radiation has been correlated with different responses. Low radiation doses seem to activate innate immune cells and fail to induce cell death. This situation develops a tumorigenic effect mediated by the cells of the immune microenvironment (17, 18). On the contrary, high radiation doses seem to induce an immunogenic effect. Schaue et al. studied tumor specific immune response in mice bearing murine melanoma irradiated with 15 Gy administered in different sizes per fraction. The authors concluded that a single dose of 7.5 Gy or higher, but not lower than 5 Gy, was immunostimulatory (19). These findings agreed with the results of Lee who compared single dose of 20 Gy against 5 Gy × 4 given over 2 weeks in a pre-clinical study. Ablative radiation of 20 Gy dramatically increased T-cell activity and tumor control, whereas the fractionated radiation showed less tumor growth inhibition (20). However, the inflammatory balance in the tumoral environment is quite complex. Radiation could promote – directly or indirectly – negative regulators such us TGF-B. It is known that latent isoform of TGFB1 is activated due to reacting oxygen species liberate the latency-associated peptide after RT (21). Furthermore, increased levels of TGF-B are detected as a consequence of M2 macrophage release after exposition to apoptotic cancer cells (22). However, until now, there is little evidence about the best radiation schedule to obtain an optimal immunogenic response.

In addition, the physical sequence of events after RT seems to be critical to mount a successful immune response. Pre-clinical models have shown that DC loaded with tumor antigens migrate toward the draining lymph nodes. This process leads to an activation of T cells that had not been previously exposed to specific tumor antigens, spreading the immune response against the tumor (20, 23). Activated CTL are guided by the chemokine gradient induced by radiation. This fact has been ascertained, for example, by the CXCL16 chemokine, which is able to recruit effector T cells to the site of the irradiated tumor area (24). In a murine model of metastatic breast cancer, the CXCL16 proved to be essential in the synergism between RT and CTLA-4 blocking (25, 26). In addition, tumor vessels have multiple barriers – through an abnormal architecture or a lower expression of endothelial adhesion molecules – that hinder the infiltration of T cells (27). At this point, RT allows the upregulation of cell adhesion molecules (CAM), which facilitates the transit of lymphocytes to tumor cells (23, 28). In a murine model of squamous cell carcinoma, blockade of CD11b – ligand for ICAM-1 – reduced the radiation-induced infiltration of myeloid cells into irradiated tumors and diminished tumor regrowth (29). VCAM-1 is up-regulated in melanoma in a process requiring IFN-γ production (28). Once the CTL are found in the tumor, radiation therapy may again influence and boost the anti-tumor immune response by the death of new tumor cells leading to the increased expression of death receptors on tumor cells such as Fas/CD95, MHC-R, CEA, NKG2D, and other co-stimulatory molecules (30–32). This fact facilitates the recognition and destruction of tumor cells by CTLs. The death of tumor cells mediated by FAS represents a mechanism independent of the T-cell receptors (TCR), so that if TCR affinity is low FAS has a potent cytotoxic role (14). Therefore, radiation modifies the characteristics of the tumor microenvironment, making it more accessible to the immune system, which supports and extends the response to RT, not only by direct and indirect injury from ionizing radiation, but also by immunomodulatory mechanisms.

The potential immunogenicity of RT is heavily influenced by the differentiation of immune cells in the tumor microenvironment. Conventional fractionated radiation therapy has traditionally been considered immunosuppressive (33). This is due, in part, to the early apoptotic death occurring in lymphocytes following low doses of radiation (34). However, lymphocyte subsets have distinct radiosensitivity. In this regard, immunosuppressive cells like macrophages are considered essentially radioresistant, whereas final effects of RT in Treg are still unclear (17, 29, 35). Results from studies by pioneering labs in this field showed that sublethal whole-body irradiation of mice bearing tumors may result in absence of responses in nude mices, and in partial or complete tumor regression in those with complete immunity. Authors considered that RT-induced tumor regression by activation of the immune response through downregulation of Treg (36, 37). However, evidence at this point is controversial, since other groups have recently postulated that Treg radioresistant behavior might lead to a percentual increase of these cells. These phenomena should deteriorate RT-induced anti-tumor immunity. In this sense, Schuler et al analyzed Treg in tumor tissues and peripheral blood of head and neck squamous cell carcinoma patients treated with chemoradiotherapy. Their results suggest that chemoradiotherapy favor survival and suppressor functions of Treg, and thus, this combinatorial approach might induce disease recurrence or even development of secondary cancers (38, 39). With respect to macrophages, local low-dose irradiation seems to induce proliferation of iNOS^+^ M1-macrophages in tumor microenvironment. These macrophages may release pro-inflammatory molecules such as TNF and facilitate cell infiltration by tumor-reactive effector T cells, improving local immune response against cancer (33). Nevertheless, it has been demonstrated that cell death after radiation may also result in M2 macrophage activation and induce immune suppression (40). The suppressive response of M2 macrophages is a key feature of inflammatory resolution, which serves to repair inflammatory destruction following control of infections by laying down supportive matrix, establishing vascular structures, and terminating adaptive immune responses (22). This change in the macrophage phenotype in tumors from M1 to M2 macrophage has been associated with early tumor growth *in vivo*. Macrophages from irradiated tumors express higher levels of iNOS, arginase-I, and COX-2, and promote tumor growth (41).

## Systemic Immune Effects of RT, the Abscopal Effect

In recent decades, RT effects in distant sites away from the original irradiated area have introduced a new concept of the highest interest, which is the ability of RT to exert systemic anti-tumoral responses. The first description of this effect was made by Robin H. Mole in 1953 (42), and this is currently denominated as the abscopal effect. The etymological definition comes from the Latin *ab* (outside) and *Scopus* (target). The abscopal effect provides new insights into the mechanisms of RT activity (43, 44).

The abscopal effect may have a dual role in the RT activity. Firstly, unwanted side events, such as the onset of inflammatory phenomena at a distance, can be generated. These side effects may produce pneumonitis or other serious phenomena like genomic instability resulting in leukemia or other neoplasms (17, 45). Secondly, the abscopal effect can have therapeutic consequences, with the reduction of distant metastasis after RT of primary tumors or localized metastases with palliative purposes as proof of principle of this phenomenon. Although proving irrefutable evidence of the abscopal effect is a difficult task, this event has been postulated in several types of tumors including melanoma, lymphoma, and hepatocellular or renal cell carcinomas (46, 47).

In the previous section of this review, it was noted that RT induces changes in the tumor microenvironment, transforming the irradiated tissue into an immunogenic hub, which serves to the immune system as a source for the identification of tumor cells. Therefore, the immune system can recognize tumor cell lines out and away from the irradiated zone. Some authors have described this “vaccine effect” by the sensitization process that generates the body, which recognizes previously unnoticed tumor cells (48). Nevertheless, the immunological mechanism underlying the abscopal effect is still unknown (49). The primary hypothesis focuses on a kind of “systemic cytokine storm” after irradiation, with the release of cytokines like TNF, IL-4, IL-18, IL-2, and GM-CSF (50–54). These cytokine may induce an anti-tumor humoral immune effect and subsequently an immune cell response against the tumor, ultimately mediated by T lymphocytes.

In the clinical setting, the abscopal effect has been studied in patients with low grade B cell lymphoma after intratumoral injection of a Toll-like receptor 9 (TLR9) agonist (CpG) during treatment with RT. Previously, authors detected the recognition and response of cytotoxic lymphocytes against B lymphoma cells *in vitro* (55). Furthermore, *in vivo* studies with murine models combining RT and immunostimulants, such as anti-CTLA antibody and the growth factor of DC (Flt-3) showed the reduction of tumor growth outside the irradiation fields (56).

To date case reports related to the abscopal effect are relatively scarce. One of the main reasons might be underdiagnosis due to the lack of knowledge of this phenomenon. As we have mentioned before, RT alone in some circumstances might develop immune response to control the upgrowth of distant metastases. Nowadays, this likelihood is getting higher, due to the addition of immune drugs to RT that might lead to a better recognition of remote tumor cell by the immune system. Nevertheless, some well-documented case reports have been described recently. In this sense, a case of regression of non-irradiated metastases from melanoma NY-ESO^+^ after receiving palliative RT combined with immunotherapy (anti-CTLA-4/ipilimumab) has been recently described by Postow et al. (57). After combined treatment (palliative RT and ipilimumab), metastatic lesions showed marked regression. Biological biomarkers were of great interest at this point with the observation of an increase of NY-ESO-1-specific antibodies, CD4^+^ ICOS high, NY-ESO-1-specific interferon-gamma-producing CD4^+^ cells and HLA-DR-expressing CD14^+^ monocytes, after RT. Simultaneously, MDSC levels decreased sharply (57). Another well described case report in a metastatic melanoma patient treated with palliative RT and immunotherapy (anti-CTLA-4/ipilimumab) has been recently published (58). In this case, the regression of non-irradiated in transit metastases after RT of the primary tumor was achieved. Again, a biomarker of immune activity was studied. In this case, autoantibodies against melanoma antigen A3 (MAGEA3) titers were measured demonstrating a systemic anti-tumor immune response (58).

The aforementioned cases serve as proof of principle for the abscopal effect theory related to the anti-tumor immune response, supporting evidences extracted from pre-clinical studies (Table 1). Therefore, association of RT and immunotherapy can open new lines of work and research in both fields. Further studies are needed to determine, which are the better RT and immunotherapy schedules and combinations to unleash this immune response against tumors.

**Table 1:** **Recompilation of case reports on abscopal effect**.

Case reports	Diagnosis	Dose RT/irradiated site	Response to RT	Associated treatments	Specific immune response markers
Antoniades et al. (74)	Stage III non-Hodgkin’s lymphoma	30 Gy in 20 fx	Regression of abdominal lymph nodes after mantle’s irradiation	No	No
Ohba et al. (50)	Metastatic hepatocellular carcinoma to bone	36 Gy to metastasis	Complete regression of the metastasis and remarkable regression of the hepatic lesions.	No	Increase of TNF-α
Wersäll et al. (73)	Metastatic renal cell carcinoma				
	Case report A: metastases in lymph nodes and lung	32 Gy in 4 fx to primary tumor	Complete regression of the lung lesions and an almost complete regression of lymph nodes	No	No
	Case report B: multiple pulmonary metastases	RT only in three pulmonary metastases (no dose mentioned)	All the metastases responded partially or completely	Thalidomide	No
	Case report C: four pulmonary metastases	30 Gy in 2 fx in two lesions in the lungs	Complete regression of treated lesions and partial regression of remaining metastatic lesions	No	No
	Case report D: metastases in lymph nodes	32 Gy in 4 fx to primary tumor	Complete response of all metastases	No	No
Okuma et al. (72)	Hepatocellular carcinoma with metastases in mediastinal lymph node and lung	60.75 Gy in 27 fx to single lung metastasis	Reduction of the mediastinal lymph node and lung metastasis unirradiated	No	No
Cotter et al. (75)	Merkel cell carcinoma with cutaneous metastases	12 Gy in 2 fx to some lesions	Treated and untreated lesions responded partially or completely	No	No
Postow et al. (57)	Metastatic melanoma with pleural-based paraspinal mass, hilar lymphadenopathy, and splenic lesions	28.5 Gy in 3 fx to pleural-based paraspinal mass	All the metastases regressed significantly	Ipilimumab	Increase of NY-ESO-1-specific antibodies, CD4^+^ ICOS high, NY-ESO-1-specific interferon-gamma-producing CD4^+^ cells and HLA-DR-expressing CD14^+^ monocytes
					Decrease of myeloid-derived-suppressor cells
Stamell et al. (58)	Metastatic melanoma	First RT: 24 Gy in 3 fx to primary tumor.	All metastases had resolved (forehead, scalp, and neck)	Ipilimumab	Increase of MAGEA3
	Development of nodal and brain mestastases	Second RT: intracranial stereotactic radiosurgery	Complete remission, including node metastasis	

## Combination of Immunotherapy and RT

Immunogenic cell death mediated by RT may serve as the basis of an effective immunogenic host response that can be modulated by other immunogenic strategies.

As previously described, synergism between RT and other immune therapies have a robust biological rationale that, related to the adaptive-cell response and generation of cytotoxic T lymphocytes, may be summarized in the following sequence of events.

(1) First signal: tumoral associated antigens availability: At this point, the effects of RT inducing an antigenic environment with the release of tumoral antigens after cell death is a fact of the highest importance since it favors the generation of an inflammatory microenvironment around the irradiated tumor. In addition, RT seems to favor antigen presentation via surface MHC-I in APCs to cytotoxic lymphocytes (59). Specifically tumor specific antigens (TSA) identification increases treatment efficacy due to immune response to selective cancer cells. Robbins et al. showed that transferring autologous lymphocytes – previously exposed to mutated cancer proteins – lead to an *in vitro* an *in vivo* tumor regression (59).
(2) Second signal: co-stimulatory/co-inhibitory molecules: Immune synapse has been revealed as a promising therapeutic target and a set of monoclonal antibodies (mAb) targeting the molecules of this virtual space is under intensive clinical research (60).
  (a) Cytotoxic T lymphocyte antigen-4 (CTLA-4): mAb anti-CTLA-4 ipilimumab has been approved by the FDA for the treatment of advanced melanoma, after demonstrating an increase in overall statistically significant survival in two randomized phase III trials in the first and second line setting (61, 62). Specifically with RT in pre-clinical models, local RT and CTLA-4 blockade have shown to mediate synergistic effects. In this sense, in mice concurrently challenged with two tumors, the treatment of one tumor with local RT in combination with the systemic administration of anti-CTLA-4 induced significant growth delay in the second tumor that did not receive local RT (63). The exact mechanism underlying the abscopal regression of unirradiated tumors has not been fully explained, but the results are consistent with an increased priming of tumor antigen-specific T cells that subsequently infiltrate the tumor. Such an effect would likely be mediated by blocking the engagement of CTLA-4 on effect or T cells in the context of intensified cross-priming capacity of DCs in the lymph nodes (48). At this point, Dewan et al. reported that a fractional dose of 8 Gy × 3 was optimal for the induction of an abscopal effect when combined with anti-CTLA-4, whereas an abscopal effect was not observed when tumors were treated with 20 Gy × 1 or 6 Gy × 5 alone or in combination with anti-CTLA-4 (63). The mechanic basis for the ability of 8 Gy × 3 to properly synergize with anti-CTLA-4 was not explored, but nevertheless the authors pointed out that this dose schedule resulted in the highest level of infiltration and IFN-γ production by T cells. The synergism between local RT and the CTLA-4 blocking observed in pre-clinical models appears to translate well into the clinic. As previously mentioned, some clinical reports in melanoma patients have demonstrated abscopal regression following treatment with local RT and anti-CTLA-4 (ipilimumab) that was associated with elevated immunity to tumor-associated antigens (58, 59).
  (b) OX40: Irradiation and anti-OX40 treatment synergistically promote infiltrating CD8^+^ T cells (64). OX40 stimulation obtains no inherent capacity to polarize T cells toward one particular effector subset, but in comparison, drives T-cell polarization in the context of the inflammatory ambient. Considering the nature of most tumor-associated antigens, it is important to observe that co-stimulation through OX40 can deliver priming of low avidity T cells and can also reverse T-cell tolerance against self-antigens. Pre-clinical and clinical data employing local ablative RT with OX40 agonistic antibody, systemic IL-2, or anti-CTLA-4 determine that signaling through CD25 and OX40 increase T-cell responses against tumor-associated antigens (64, 65). Future clinical trials involving local RT, anti-CTLA-4, and agonistic OX40 are promising and may hopefully induce impressive results.
  (c) Programed death ligand 1 (PD-L1): Evidence in pre-clinical models suggests that a PD-L1 blockade is essential in some situations to fully uncover anti-tumor immunity that is induced by local RT in combination with co-stimulatory receptor engagement. Local RT combined with anti-OX40 and anti-PD-L1 has shown to mediate complete regression in orthotopic AT-3 mammary tumors. (66).
  (d) CD137: Agonist antibodies to CD137 and CD137-ligand co-stimulate T cells after TCR stimulation (67). Anti-CD137 mAb immunotherapy has been combined with RT in pre-clinical models with encouraging results. At this point, mAb to CD137 combined with a hypofractioned RT schedule induced up to a 100% rejection rate of orthotopically implanted triple negative mammary tumors (66).
  Besides CTLA-4, OX40, PD-1, and CD137, other co-stimulatory and co-inhibitory molecules like CD40 or glucocorticoid induced TNFR (GITR) represent other new emerging targets for immunotherapy (68).
(3) Third signal: cytokines: To complete the process of a full effector T-cell response with clonal expansion and differentiation on memory T cells, a third signal provided by cytokines is needed (69). In this step, cytokines that mediate and amplify adaptive immune responses are produced mainly by antigen-stimulated T lymphocytes, and they include, among others, IL-2, IL-4, IL-5, IL-13, and IFN-γ (70). Colony stimulating factors (CSFs) are cytokines made by activating T cells, macrophages, endothelial cells, and bone marrow stem cells that stimulate the growth of bone marrow progenitors, thereby providing a source of additional inflammatory cytokines (69). GM-CSF is one of the most important cytokines in cancer microenvironment and among their functions it may induce the maturation of DCs enabling the priming of immune responses and the generation of memory T cells (15). Some preliminary data support an eventual synergism between cytokines and RT. Demaria et al. designed a trial that recruited metastatic cancer patients that were previously treated with chemotherapy and afterward with RT to one metastatic lesion coupled with GM-CSF s.c for 14 days. Although it was a heavily pre-treated population, a response outside of the RT field was detected in 4 out of 12 patients, serving as proof of principle of the eventual abscopal effect induced by RT and cytokines (71).

A tumor microenvironment is a challenging battlefield where many actors interact. From a clinical point of view, synergy between RT and immune therapies open a new breakthrough for clinical research. Specifically, targeting of immunostimulatory and inhibitory checkpoints with immunomodulatory mAbs can “awake” and promote the systemic effects induced by RT that ultimately may be maintained and boosted by cytokines.

Monitoring immune response generated after RT is another big challenge in this field. A set of different biomarkers in blood and in tissue may aid in detecting the “switching-on” of the immune response, if it finally occurs, and to follow its “real-time” evolution. This may provide valuable information in order to amplify and boost (with cytokines and other strategies) the immune responses detected. Clinical development of anti-CTLA-4 mAb in the last few years has increased the interest in the search and validation of immune biomarkers. At this point, characterization of antigenic-specific immune responses has been performed for several cancer related antigens. Serological and T-cell responses to NY-ESO-1 and MAGEA3 have been detected and prove to be useful to monitor immune responses and clinical evolution in patients with melanoma treated with ipilimumab and with the combination of RT and ipilimumab (57, 58). Some subsets of immunosuppressive cells like Treg, TAM, and MDSC may represent other interesting biomarkers. Furthermore, in the abscopal case reported by Postow et al. with ipilimumab and RT, a decline in the levels of MDSC (CD14 + HLA-DR^low^) was ascertained after RT and these findings were timely correlated with the clinical response detected (57).

Every clinical trial with the aim of studying the synergism between RT and immunotherapy in the future might introduce some of the biomarkers previously cited (or a combination of them) in order to detect accurately if the clinical outcomes eventually observed are related or not to the combination approach. This is a critical point to truly confirm the abscopal effect hypothesis, as noted in some case reports illustrated in Table 1 (50, 57, 58, 71–75). Furthermore, these biological markers may represent powerful tools to increase not only the quality (duration of responses) but also the quantity of life of cancer patients who will benefit from this approach.

## Conclusion

The data available support the hypothesis of a mediated immune anti-tumor activity for RT. Our understanding of this effect at the molecular level has substantially increased in the last few years, and a couple of well-documented clinical cases have been reported recently, which serve as proof of principle of the abscopal effect in the clinical scenario. Therefore, combined strategies of radio-immunotherapy will eventually modulate immune response toward cancer cell destruction leading to meaningful clinical results. Nowadays, several clinical trials are ongoing exploring the immune consequences of RT, especially with immune checkpoints, and they will probably shed more light to this topic. Interestingly, validation of biomarkers like antibodies against NY-ESO-1 and MAGEA3 or measurement of TAM and MDSC is another very important task in order to facilitate the design of fine tune approaches related to these new immunotherapies and combinations in the coming future.

## Conflict of Interest Statement

The authors declare that the research was conducted in the absence of any commercial or financial relationships that could be construed as a potential conflict of interest.

## References

[1] Formenti SC Demaria S Systemic effects of local RT. Lancet Oncol (2009) 10:718–26 10.1016/S1470-2045(09)70082-8 19573801 PMC2782943

[2] Stone HB Peters LJ Milas L Effect of host immune capability on radiocurability and subsequent transplantability of a murine fibrosarcoma. J Natl Cancer Inst (1979) 63:1229–35 291749

[3] Steel GG McMillan TJ Peacock JH The 5Rs of radiobiology. Int J Radiat Biol (1989) 56:1045–8 10.1080/09553008914552491 2574214

[4] Demaria S Formenti SC Radiation as an immunological adjuvant: current evidence on dose and fractionation. Front Oncol (2012) 2:153 10.3389/fonc.2012.00153 23112958 PMC3481113

[5] Demaria S Formenti SC Sensors of ionizing radiation effects on the immunological microenvironment of cancer. Int J Radiat Biol (2007) 83:819–25 10.1080/09553000701481816 17852561

[6] Shiao SL Coussens LM The tumor-immune microenvironment and response to radiation therapy. J Mammary Gland Biol Neoplasia (2010) 15:411–21 10.1007/s10911-010-9194-9 21161342 PMC3011087

[7] Demaria S Bhardwaj N McBride WH Formenti SC Combining RT and immunotherapy: a revived partnership. Int J Radiat Biol (2005) 63:655–6 10.1016/j.ijrobp.2005.06.032 16199306 PMC1489884

[8] Matzinger P Tolerance, danger, and the extended family. Annu Rev Immunol (1994) 12:991–1045 10.1146/annurev.immunol.12.1.991 8011301

[9] Tesniere A Apetoh L Ghiringhelli F Joza N Panaretakis T Kepp O Immunogenic cancer cell death: a key-lock paradigm. Curr Opin Immunol (2008) 20:504–11 10.1016/j.coi.2008.05.007 18573340

[10] Obeid M Ecto-calreticulin in immunogenic chemotherapy. Immunol Rev (2007) 220:22–34 10.1111/j.1600-065X.2007.00567.x 17979837

[11] Apetoh L Ghiringhelli F Tesniere A Obeid M Ortiz C Criollo A Toll-like receptor 4-dependent contribution of the immune system to anticancer chemotherapy and RT. Nat Med (2007) 13:1050–9 10.1038/nm1622 17704786

[12] Gasser S Orsulic S Brown EJ Raulet DH The DNA damage pathway regulates innate immune system ligands of the NKG2D receptor. Nature (2005) 436:1186–90 10.1038/nature03884 15995699 PMC1352168

[13] Kim JY Son YO Park SW Bae JH Chung JS Kim HH Increase of NKG2D ligands and sensitivity to NK cell-mediated cytotoxicity of tumor cells by heat shock and ionizing radiation. Exp Mol Med (2006) 38:474–474 10.1038/emm.2006.56 17079863

[14] Kessler B Hudrisier D Schroeter M Tschopp J Cerottini JC Luescher IF Peptide modification or blocking of CD8, resulting in weak TCR signaling, can activate CTL for Fas- but not perforin-dependent cytotoxicity or cytokine production. J Immunol (1998) 161:6939–46 9862728

[15] Larsson M Fonteneau JF Bhardwaj N Dendritic cells resurrect antigens from dead cells. Trends Immunol (2001) 22:141–8 10.1016/S1471-4906(01)01860-9 11286729

[16] Skoberne M Beignon AS Bhardwaj N Danger signals: a time and space continuum. Trends Mol Med (2004) 10:251–7 10.1016/j.molmed.2004.04.001 15177188

[17] Coates PJ Rundle JK Lorimore SA Wright EG Indirect macrophage responses to ionizing radiation: implications for genotype-dependent bystander signaling. Cancer Res (2008) 68:450–6 10.1158/0008-5472.CAN-07-3050 18199539

[18] Wright EG Coates PJ Untargeted effects of ionizing radiation: implications for radiation pathology. Mutat Res (2006) 597:119–32 10.1016/j.mrfmmm.2005.03.035 16438994

[19] Schaue D Ratikan JA Iwamoto KS McBride WH Maximizing tumor immunity with fractionated radiation. Int J Radiat Oncol Biol Phys (2012) 83:1306–10 10.1016/j.ijrobp.2011.09.049 22208977 PMC3337972

[20] Lee Y Auh SL Wang Y Burnette B Wang Y Meng Y Therapeutic effects of ablative radiation on local tumor require CD8+ T cells: changing strategies for cancer treatment. Blood (2009) 114:589–95 10.1182/blood-2009-02-206870 19349616 PMC2713472

[21] Jobling MF Mott JD Finnegan MT Jurukovski V Erickson AC Walian PJ Isoform-specific activation of latent transforming growth factor beta (LTGF-beta) by reactive oxygen species. Radiat Res (2006) 166:839–48 10.1667/RR0695.1 17149983

[22] Crittenden MR Cottam B Savage T Nguyen C Newell P Gough MJ Expression of NF-κB p50 in tumor stroma limits the control of tumors by radiation therapy. PLoS One (2012) 7:e39295 10.1371/journal.pone.0039295 22761754 PMC3386283

[23] Lugade AA Moran JP Gerber SA Rose RC Frelinger JG Lord EM Local radiation therapy of B16 melanoma tumors increases the generation of tumor antigen-specific effector cells that traffic to the tumor. J Immunol (2005) 174:7516–23 15944250 10.4049/jimmunol.174.12.7516

[24] Matsumura S Demaria S Up-regulation of the pro-inflammatory chemokine CXCL16 is a common response of tumor cells to ionizing radiation. Radiat Res (2010) 173:418–25 10.1667/RR1860.1 20334513 PMC2857712

[25] Matsumura S Wang B Kawashima N Braunstein S Badura M Cameron TO Radiation-induced CXCL16 release by breast cancer cells attracts effector T cells. J Immunol (2008) 181:3099–107 18713980 10.4049/jimmunol.181.5.3099 PMC2587101

[26] Demaria S Kawashima N Yang AM Devitt ML Babb JS Allison JP Immune-mediated inhibition of metastases following treatment with local radiation and CTLA-4 blockade in a mouse model of breast cancer. Clin Cancer Res (2005) 11:728–34 15701862

[27] Chen Q Wang WC Evans SS Tumor microvasculature as abarrier to antitumor immunity. Cancer Immunol Immunother (2003) 52:670–9 10.1007/s00262-003-0425-4 12920482 PMC11032784

[28] Lugade AA Sorensen EW Gerber SA Moran JP Frelinger JG Lord EM Radiation-induced IFN-gamma production with in the tumor microenvironment influences anti-tumor immunity. J Immunol (2008) 180:3132–9 18292536 10.4049/jimmunol.180.5.3132

[29] Ahn GO Tseng D Liao CH Dorie MJ Czechowicz A Brown JM Inhibition of Mac-1 (CD11b/CD18) enhances tumor response to radiation by reducing myeloid cell recruitment. Proc Natl Acad Sci U S A (2010) 107:8363–8 10.1073/pnas.0911378107 20404138 PMC2889597

[30] Chakraborty M Abrams SI Coleman CN Camphausen K Schlom J Hodge JW External beam radiation of tumors alters phenotype of tumor cells to render them susceptible to vaccine-mediated T-cell killing. Cancer Res (2004) 64:4328–37 10.1158/0008-5472.CAN-04-0073 15205348

[31] Sheard MA Ionizing radiation as a response-enhancing agent for CD95-mediated apoptosis. Int J Cancer (2001) 96:213–20 10.1002/ijc.1020 11474495

[32] Friedman EJ Immune modulation by ionizing radiation and its implications for cancer immunotherapy. Curr Pharm Des (2002) 8:1765–80 10.2174/1381612023394089 12171547

[33] Klug F Prakash H Huber PE Seibel T Bender N Halama N Low-dose irradiation programs macrophage differentiation to an iNOS(+)/M1 phenotype that orchestrates effective T cell immunotherapy. Cancer Cell (2013) 24:589–602 10.1016/j.ccr.2013.09.014 24209604

[34] Belka C Ottinger H Kreuzfelder E Weinmann M Lindemann M Lepple-Wienhues A Impact of localized RT on blood immune cells counts and function in humans. Radiother Oncol (1999) 50:199–204 10.1016/S0167-8140(98)00130-3 10368044

[35] DeNardo DG Brennan DJ Rexhepaj E Ruffell B Shiao SL Madden SF Leukocyte complexity predicts breast cancer survival and functionally regulates response to chemotherapy. Cancer Discovery (2011) 1:54–67 10.1158/2159-8274.CD-10-0028 22039576 PMC3203524

[36] Hellstrom KE Hellstrom I Kant JA Tamerius JD Regression and inhibition of sarcoma growth by interference with a radiosensitive T-cell population. J Exp Med (1978) 148:799–804 10.1084/jem.148.3.799 308987 PMC2184991

[37] North RJ Radiation-induced, immunologically mediated regression of an established tumor as an example of successful therapeutic immunomanipulation. Preferential elimination of suppressor T cells allows sustained production of effector T cells. J Exp Med (1986) 164:1652–66 10.1084/jem.164.5.1652 2945892 PMC2188446

[38] Schaue D Xie MW Ratikan JA McBride WH Regulatory T cells in radiotherapeutic responses. Front Oncol (2012) 2:90 10.3389/fonc.2012.00090 22912933 PMC3421147

[39] Schuler PJ Harasymczuk M Schilling B Saze Z Strauss L Lang S Effects of adjuvant chemoRT on the frequency and function of regulatory T cells in patients with head and neck cancer. Clin Cancer Res (2013) 19:6585–96 10.1158/1078-0432.CCR-13-0900 24097865 PMC3855337

[40] Chiang CS Fu SY Wang SC Yu CF Chen FH Lin CM Irradiation promotes an M2 macrophage phenotype in tumor hypoxia. Front Oncol (2012) 6(2):89 10.3389/fonc.2012.00089 22888475 PMC3412458

[41] Tsai CS Chen FH Wang CC Huang HL Jung SM Wu CJ Macrophages from irradiated tumors express higher levels of iNOS, arginase-I and COX-2, and promote tumor growth. Int J Radiat Oncol Biol Phys (2007) 68:499–507 10.1016/j.ijrobp.2007.01.041 17398016

[42] Mole RJ Whole body irradiation – radiology or medicine? Br J Radiol (1953) 26:234–41 10.1259/0007-1285-26-305-234 13042090

[43] Trosko JE Chang CC Upham BL Tai MH Low-dose ionizing radiation: Induction of differential intracellular signaling possibly affecting intercellular communication. Radiat Environ Biophys (2005) 44:3–9 10.1007/s00411-005-0269-8 15821925

[44] Kaminski JM Shinohara E Summers JB Niermann KJ Morimoto A Brousal J The controversial abscopal effect. Cancer Treat Rev (2005) 31:159–72 10.1016/j.ctrv.2005.03.004 15923088

[45] Lorimore SA Chrystal JA Robinson JI Coates PJ Wright EG Chromosomal instability in unirradiated hemaopoietic cells induced by macrophages exposed in vivo to ionizing radiation. Cancer Res (2008) 68:8122–6 10.1158/0008-5472.CAN-08-0698 18829571

[46] Kingsley DP An interesting case of possible abscopal effect in malignant melanoma. Br J Radiol (1975) 48:863–6 10.1259/0007-1285-48-574-863 811297

[47] Robin HI AuBuchon J Varanasi VR Weinstein AB The abscopal effect: demonstration in lymphomatous involvement of kidneys. Med Pediatr Oncol (1981) 9:473–6 10.1002/mpo.2950090510 7029238

[48] Formenti SC Demaria S Radiation therapy to convert the tumor into an in situ vaccine. Int J Radiat Biol (2012) 84:879–80 10.1016/j.ijrobp.2012.06.020 PMC3811126 23078897

[49] Camphausen K Moses MA Ménard C Sproull M Beecken WD Folkman J Radiation abscopal antitumor effect is mediated through p53. Cancer Res (2003) 63:1990–3 12702593

[50] Ohba K Omagari K Nakamura T Ikuno N Saeki S Matsuo I Abscopal regression of hepatocellular carcinoma after RT for bone metastasis. Gut (1998) 43:575–7 10.1136/gut.43.4.575 9824589 PMC1727260

[51] Moret-Tatay I Díaz J Marco FM Crespo A Aliño SF Complete tumor prevention by engineered tumor cell vaccines employing nonviral vectors. Cancer Gene Ther (2003) 10:887–97 10.1038/sj.cgt.7700646 14712315

[52] Tatsumi T Huang J Gooding WE Gambotto A Robbins PD Vujanovic NL Intratumoral delivery of dendritic cells engineered to secrete both interleukin (IL)-12 and IL-18 effectively treats local and distant disease in association with broadly reactive Tc1-type immunity. Cancer Res (2003) 63:6378–86 14559827

[53] Hillman GG Slos P Wang Y Wright JL Layer A De Meyer M Tumor irradiation followed by intratumoral cytokine gene therapy for murine renal adenocarcinoma. Cancer Gene Ther (2004) 11:61–72 10.1038/sj.cgt.7700656 14681727

[54] Nakanishi M Chuma M Hige S Asaka M Abscopal effect on hepatocellular carcinoma. Am J Gastroenterol (2008) 103:1320–1 10.1111/j.1572-0241.2007.01782_13.x 18477367

[55] Brody JD Ai WZ Czerwinski DK Torchia JA Levy M Advani RH In situ vaccination with a TLR9 agonist induces systemic lymphoma regression: a phase I/II study. J Clin Oncol (2010) 28:4324–32 10.1200/JCO.2010.28.9793 20697067 PMC2954133

[56] Demaria S Ng B Devitt ML Babb JS Kawashima N Liebes L Ionizing radiation inhibition of distant untreated tumors (abscopal effect) is immune mediated. Int J Radiat Biol (2004) 58:862–70 10.1016/j.ijrobp.2003.09.012 14967443

[57] Postow MA Callahan MK Barker CA Yamada Y Yuan J Kitano S Immunologic correlates of the abscopal effect in a patient with melanoma. N Engl J Med (2012) 366:925–31 10.1056/NEJMoa1112824 22397654 PMC3345206

[58] Stamell EF Wolchok JD Gnjatic S Lee NY Brownell I The abscopal effect associated with a systemic anti-melanoma immune response. Int J Radiat Biol (2013) 2:293–5 10.1016/j.ijrobp.2012.03.017 22560555 PMC3415596

[59] Robbins PF Lu YC El-Gamil M Li YF Gross C Gartner J Mining exomic sequencing data to identify mutated antigens recognized by adoptively transferred tumor-reactive T cells. Nat Med (2013) 19:747–52 10.1038/nm.3161 23644516 PMC3757932

[60] de la Cruz-Merino L Grande-Pulido E Albero-Tamarit A Codes-Manuel deVillena ME Cancer and immune response: old and new evidence for future challenges. Oncologist (2008) 13:1246–54 10.1634/theoncologist.2008-0166 19056856

[61] Hodi FS O’Day SJ McDermott DF Weber RW Sosman JA Haanen JB Improved survival with ipilimumab in patients with metastatic melanoma. N Engl J Med (2010) 363:711–23 10.1056/NEJMoa1003466 20525992 PMC3549297

[62] Robert C Thomas L Bondarenko I O’Day S M DJW Garbe C Ipilimumab plus dacarbazine for previously untreated metastatic melanoma. N Engl J Med (2011) 364:2517–26 10.1056/NEJMoa1104621 21639810

[63] Dewan MZ Galloway AE Kawashima N Dewyngaert JK Babb JS Formenti SC Fractionated but not single-dose RT induces an immune-mediated abscopal effect when combined with anti-CTLA-4 antibody. Clin Cancer Res (2009) 15:5379–88 10.1158/1078-0432.CCR-09-0265 19706802 PMC2746048

[64] Gough MJ Crittenden MR Sarff M Pang P Seung SK Vetto JT Adjuvant therapy with agonistic antibodies to CD134 (OX40) increases local control after surgical or radiation therapy of cancer in mic. J Immunother (2010) 33:798–809 10.1097/CJI.0b013e3181ee7095 20842057 PMC3563298

[65] Jensen SM Maston LD Gough MJ Ruby CE Redmond WL Crittenden M Signaling through OX40 enhances antitumor immunity. Semin Oncol (2010) 37:524–32 10.1053/j.seminoncol.2010.09.013 21074068 PMC2997672

[66] Deng L Liang H Burnette B Beckett M Darga T Weichselbaum RR Irradiation and anti-PD-L1 treatment synergistically promote antitumor immunity in mice. J Clin Investiga (2014) 124:687–95 10.1172/JCI67313 24382348 PMC3904601

[67] Sabbagh L Pulle G Liu Y Tsitsikov EN Watts TH ERK-dependent Bim modulation downstream of the 4-1BB-TRAF1 signaling axis is a critical mediator of CD8 T cell survival in vivo. J Immunol (2008) 180:8093–101 18523273 10.4049/jimmunol.180.12.8093

[68] Melero I Grimaldi AM Perez-Gracia JL Ascierto PA Clinical development of immunostimulatory monoclonal antibodies and opportunities for combination. Clin Cancer Res (2013) 19:997–1008 10.1158/1078-0432.CCR-12-2214 23460531

[69] Abbas AK Lichtman AH Pillai S Immunity to tumors. In: Abbas AK Lichtman AH Pillai S, editors. Cellular and Molecular Immunology. 6th Ed Philadelphia: Saunders Elsevier (2007). p. 397–417

[70] Ribas A Butterfield LH Glaspy JA Economou JS Current developments in cancer vaccines and cellular immunotherapy. J Clin Oncol (2003) 21:2415–32 10.1200/JCO.2003.06.041 12805342

[71] Formenti SC Friedman K Chao K Adams S Fenton-Kerimian M Donach ME Abscopal response in irradiated patients: results of a proof of principle trial. Int J Radiat Oncol (2008) 72:S6–7 10.1016/j.ijrobp.2008.06.782

[72] Okuma K Yamashita H Niibe Y Hayakawa K Nakagawa K Abscopal effect of radiation on lung metastases of hepatocellular carcinoma: a case report. J Med Case Rep (2011) 5:111 10.1186/1752-1947-5-111 21418591 PMC3069951

[73] Wersäll PJ Blomgren H Pisa P Lax I Kälkner K-M Svedman C Regression of non-irradiated metastases after extracranial stereotactic RT in metastatic renal cell carcinoma. Acta Oncol (2006) 45:493–7 10.1080/02841860600604611 16760190

[74] Antoniades J Brady LW Lightfoot DA Lymphangiographic demonstration of the abscopal effect in patients with malignant lymphomas. Int J Radiat Oncol (1977) 2:141–7 10.1016/0360-3016(77)90020-7 403163

[75] Cotter SE Dunn GP Collins KM Sahni D Zukotynski KA Hansen JL Abscopal effect in a patient with metastatic Merkel cell carcinoma following radiation therapy: potential role of induced antitumor immunity. Arch Dermatol (2001) 147:870–2 10.1001/archdermatol.2011.176 21768497

